# Transient Receptor Potential (TRP) Ion Channels Involved in Malignant Glioma Cell Death and Therapeutic Perspectives

**DOI:** 10.3389/fcell.2021.618961

**Published:** 2021-08-12

**Authors:** Florence Lefranc

**Affiliations:** Department of Neurosurgery, Hôpital Erasme, Université Libre de Bruxelles, Brussels, Belgium

**Keywords:** malignant glioma, cell death, TRP ion channels, treatment, cannabidiol

## Abstract

Among the most biologically, thus clinically, aggressive primary brain tumors are found malignant gliomas. Despite recent advances in adjuvant therapies, which include targeted and immunotherapies, after surgery and radio/chemotherapy, the tumor is recurrent and always lethal. Malignant gliomas also contain a pool of initiating stem cells that are highly invasive and resistant to conventional treatment. Ion channels and transporters are markedly involved in cancer cell biology, including glioma cell biology. Transient receptor potential (TRP) ion channels are calcium-permeable channels implicated in Ca^2+^ changes in multiple cellular compartments by modulating the driving force for Ca^2+^ entry. Recent scientific reports have shown that these channels contribute to the increase in glioblastoma aggressiveness, with glioblastoma representing the ultimate level of glioma malignancy. The current review focuses on each type of TRP ion channel potentially involved in malignant glioma cell death, with the ultimate goal of identifying new therapeutic targets to clinically combat malignant gliomas. It thus appears that cannabidiol targeting the TRPV2 type could be such a potential target.

## Introduction

### Malignant Glioma Generality

Among the most common malignant primary tumors are encountered malignant gliomas, which are associated with dismal prognosis. Precise statistics from the United States report for example 17,000 new diagnoses in 2017 (Ostrom et al., 2017). These tumors are characterized by extensive proliferation, invasion, migration, angiogenesis, immunosuppression, and resistance to conventional treatment (Lefranc et al., 2018; Locarno et al., 2020). Malignant gliomas include grade II (gliomas), III (anaplastic gliomas), and IV (glioblastoma) tumors. The median survival of glioblastoma is only 16 months because of the high rate of tumor recurrence (>95%) (Lefranc et al., 2018), even under aggressive treatment, including large surgical resection followed by combined radio- and temozolomide chemotherapy and adjuvant chemotherapy with the same compound (Stupp et al., 2009). This high rate of tumor recurrence is linked to the dramatic infiltrative properties of glioma cells into the brain parenchyma, rendering therefore elusive curative surgical resection as well as conventional treatments using genotoxic radiotherapy and cytotoxic chemotherapy, and even antiangiogenic therapies (Wick et al., 2017). Targeted therapies and immunotherapies also failed in efficaciously combating malignant gliomas (McGranahan et al., 2019).

Heterogeneous populations of tumor-differentiated cells coexisting with subpopulations displaying stem cell properties are present in glioblastomas. The marked biological, thus clinical, aggressiveness of glioblastoma stem cells (GSCs) relates among others to their dramatic invasive nature into the brain parenchyma, high level of mobility into the brain parenchyma, and high resistance to both radio- and chemotherapy. GSCs have also the capacity to self-renew and are now known to be directly responsible for the recurrence and clinical relapse of glioblastomas (Colwell et al., 2017; Matarredona and Pastor, 2019).

All grade II gliomas have the tendency to transform into more aggressive grade III (anaplastic) or even grade IV gliomas (secondary glioblastoma); likewise, grade III gliomas can similarly transform into grade IV (secondary glioblastoma).

A glioblastoma is a mosaic of various cell populations associated with distinct dynamic cell states as recently revealed by genome-wide sequencing, and this dramatic cell heterogeneity within a given glioblastoma renders any type of treatment very difficult (Sottoriva et al., 2013; Patel et al., 2014; Bernstock et al., 2019).

### Ion Channels Involved in Malignant Glioma Progression

Ion channels are classified by ion selectivity (sodium channels, potassium channels, chloride channels, proton channels, unselective channels, etc.), gating mechanism (voltage-gated, ligand-gated, cyclic nucleotide-gated, light-gated, and mechanosensitive), or localization (plasma membrane or intracellular) (Alexander et al., 2019). These channels display marked roles in a plethora of cellular processes and in cancer progression (Becchetti et al., 2013; Litan and Langhans, 2015; Prevarskaya et al., 2018).

Several ion channels are implicated in malignant glioma proliferation, migration, invasion, and cell death. For example, genome-wide analyses of glioblastoma revealed that, of 555 genes involved in potassium, sodium, chloride, calcium channels, and other ion transport, 55 mutations were detected, affecting 90% of the glioblastoma samples studied (Parsons et al., 2008).

It has been experimentally demonstrated already two decades ago that glioma cells invading the brain parenchyma must modify their shape and/or volume to perform their invasive journey (Soroceanu et al., 1999). Shape-volume changes in glioma cells are mediated, at least partly, by chloride currents, which, while affecting net salt fluxes across glioma cell membranes, induce water efflux, resulting in turn in glioma cell shrinkage facilitating their migration through minute extracellular spaces of the brain (Ransom et al., 2001; Habela et al., 2009).

In gliomas, cell condensation is a hallmark of intrinsic and extrinsic apoptosis and requires the concerted activation of chloride- and calcium-activated potassium channels, leading to the loss of water (Ernst et al., 2008). We previously reviewed the implications of the roles of potassium channels in glioma progression and migration, e.g., Kv1.3 and Kv1.5, Kv10.1, Kv11.1, KCa1.1, and KCa3.1 (Lefranc et al., 2012, 2018).

Morrone et al. (2016) reviewed the role of calcium channels in malignant brain tumor therapy.

**FIGURE 1:**
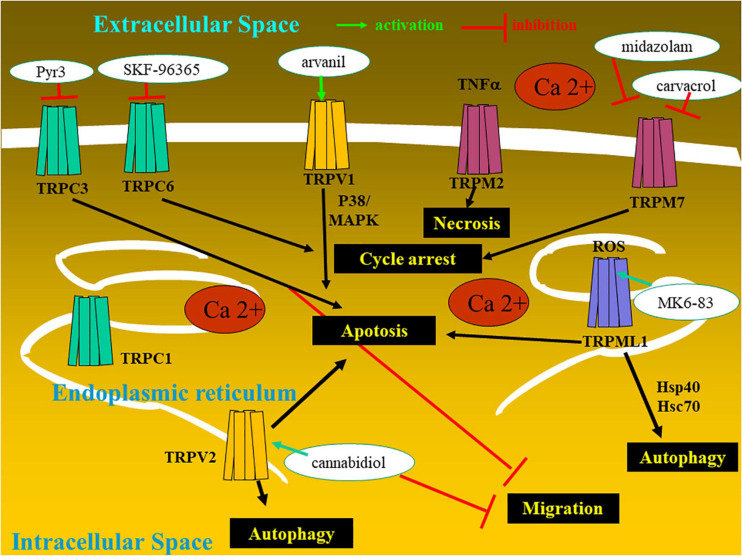
Glioma cell with the expression of transient receptor potential (TRP) ion channels and the therapeutic potentialities.

The present review focuses on transient receptor potential (TRP) calcium channels, which modulate the driving force for Ca^2+^ entry from extra- into intracellular compartments (Figure 1). For each type of TRP ion channel described below, we focused our attention in identifying specific TRP channels involved in glioma cell death, rendering them as potential new therapeutic targets to combat general malignant gliomas, with a particular focus on glioblastoma (Table 1).

**TABLE 1:** TRP ion channels involved in malignant glioma cell death and therapeutic perspectives.

TRP channels		Prognostic marker	Glioma cells *in vitro*				Experimental glioma *in vivo*		Potential therapeutic compounds (figure 2)	Clinical data	References
				
			Proliferation	Cell death	Migration/invasion	Chemo-sensitivity	Growth	Survival			
TRPC	C1	Yes	Decreased (shRNA)	–	–	–	Decreased (shRNA)	–	–	–	Bomben and Sontheimer, 2010
	C3	Yes	Decreased	Apoptosis	Decreased	–	Decreased		Pyr3 (antagonist)	–	Chang et al., 2018
	C5	–	–	–	–	–	–	–	Riluzole (agonist) Prednisolone (agonist)	–	Richter et al., 2014; Beckmann et al., 2017
	C6	Yes	–	Cell cycle arrest G2/M	–	–	Decreased	Increased	SKF-96365 (antagonist)	–	Ding et al., 2010; Song et al., 2014
TRPV	V1	Yes	–	Apoptosis endoplasmic reticulum stress	–	–	–	–	Arvanil (agonist)	–	Amantini et al., 2007; Stock et al., 2012
	V2	Yes	Decreased in hGBM cells and in GSCs	Apoptosis autophagy	Decreased	Increased	Decreased	Increased	Cannabidiol (agonist) delta9-THC (agonist)	Phase I (Dall’Stella et al., 2019 and Likar et al., 2019) Phase II trial (mixture of cannabidiol and delta9-THC) (Schultz and Beyer, 2017) Phase I trial (Guzmán et al., 2006; Velasco et al., 2007)	Marcu et al., 2010; Nabissi et al., 2010, 2013, 2015; Torres et al., 2011; Morelli et al., 2012; Ladin et al., 2016; López-Valero et al., 2018; Santoni et al., 2020a,b
TRPML	ML1	Yes	–	Autophagy apoptosis	–	–	–	–	MK6-83 (agonist)	–	Morelli et al., 2019
	ML2	Yes	Decreased (siRNA)	Apoptosis	–	–	–	–	–	–	Morelli et al., 2016
TRPM	M2	Yes	Decreased	Increased (TRPM2 transfection)	Decreased	–	–	–	–	–	Ishii et al., 2007; Bao et al., 2020
	M7	Yes	Decreased (siRNA and antagonist)		Decreased (antagonist)	–	–	–	Carvacrol (antagonist) Midazolam (antagonist)	–	Chen et al., 2015, 2016; Leng et al., 2015
	M8	Yes	–	Apoptosis repressed	Stimulated	Radio resistance	–	–	Antibodies	–	Klumpp et al., 2017

### Malignant Glioma Cell Death

Glioma, melanoma, non-small cell lung cancer, and esophageal cancer, among others, are resistant to proapoptotic stimuli and are typically associated with dismal prognoses (Hanahan and Weinberg, 2011; Kornienko et al., 2013) and display therefore resistance to conventional cytotoxic pro-apoptotic drugs. Cytotoxic compounds that induce non-apoptotic cellular mechanisms, such as necrosis, senescence, autophagy, and mitotic catastrophe, are of great hopes to combat these cancer types displaying various levels of resistance to pro-apoptotic stimuli (Tang et al., 2019). The readers interested by precise definitions about the various cell death types should refer to the nomenclature established by Kroemer and colleagues (Galluzzi et al., 2012). The authors distinguished 13 distinct cell death types (Galluzzi et al., 2012), to which we have added methuosis, paraptosis, oncosis, and lysosomal membrane permeabilization (LMP) cell death types (Kornienko et al., 2013).

**FIGURE 2:**
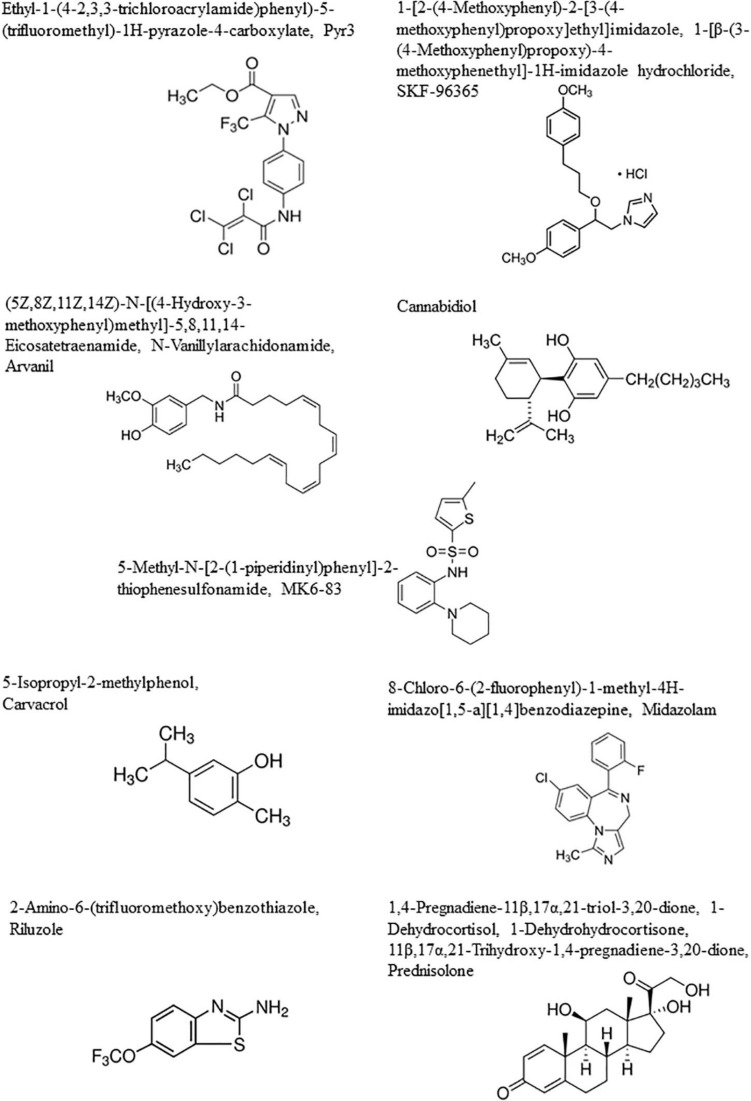
Chemical structures.

In the current review, the subchapter entitled therapeutic perspective aims to analyze the types of TRP-targeting drugs that could be of help in overcoming the resistance of glioma cancer cells and glioma stem cells to conventional therapies (Figure 2 and Table 1).

## Modulating Transient Receptor Potential Channels for Inducing Cell Death in Malignant Glioma

### Transient Receptor Potential Ion Channels

The mammalian TRP channel superfamily encompasses 28 identified members of Ca^2+^-permeable channels, with diverse physiological functions and cellular distributions (Ramsey et al., 2006; Venkatachalam and Montell, 2007; Nilius and Owsianik, 2011). TRP channels can be localized on the plasma membrane or in intracellular membranes and are involved in numerous fundamental cell functions (Nilius and Owsianik, 2011). Based on their structural homology and function, TRP channels are grouped into seven subfamilies in mammals: TRPC (canonical), TRPM (melastatin), TRPV (vanilloid), TRPP (polycystin), TRPA (ankyrin-like), TRPML (mucolipin), and TRPN (Drosophila NOMPC) (Montell et al., 2002; Li, 2017; Table 1). All TRP channels contain six transmembrane segments and a pore-forming loop between the 5th and 6th segments (Nilius and Owsianik, 2011). As TRPs are non-selective cation channels, their effects can be attributed to K^+^ and Na^+^ flux, but the role of Ca^2+^ is the most studied. TRP channels are non-selective Ca^2+^-permeable cation channels, with the exception of TRPM4 and TRPM5, which are Ca^2+^-impermeable. Some hallmarks of cancer pathophysiology are associated with the dysregulation of multiple downstream Ca^2+^-homeostasis-related effectors, a fact that explains why TRP channels are actually involved in the regulation versus dysregulation of growth, proliferation, migration, and invasion of cancer cells, including melanoma; prostate, breast, kidney, and bladder carcinomas; and gliomas (Bodding, 2007; Prevarskaya et al., 2007; Gkika and Prevarskaya, 2009; Chen et al., 2014; Bernardini et al., 2015; Santoni et al., 2020a,b; Yang and Kim, 2020).

### Transient Receptor Potential Ion Channels in Malignant Glioma Cell Death

Among the TRP channels already identified in glioma cells with demonstrated regulatory effects on migration, proliferation, and apoptosis, let us cite TRPC1, TRPC3, TRPC6, TRPV1, TRPV2, TRPML 1 and 2, TRPM2, TRPM7, and TRPM8 (Alptekin et al., 2015; Morelli et al., 2016, 2019; Chang et al., 2018; Figure 1 and Table 1).

#### TRPC Channels

TRPCs relate to the canonical family, which includes seven members that assemble as homo- or heterotetramers (Putney, 2005; Schaefer, 2005). TRPC channels may be activated directly by diacylglycerol (Kress et al., 2008) or indirectly through calcium release from the endoplasmic reticulum following stimulation of the inositol triphosphate receptor (Sours-Brothers et al., 2009). We report below only those TRPC channels for which roles have been evidenced in glioma cell biology.

##### TRPC1

TRPC1 (subfamily C member 1) appears to be important for cytokinesis in cell proliferation and migration (Nesin and Tsiokas, 2014) and angiogenesis. TRPC1–lipid raft complexes are essential for certain stimulus-induced chemotaxis as it is for example the case with epidermal growth factors (Bomben et al., 2011).

Human malignant gliomas contain Ca^2+^-permeable TRPC1 channels as evidenced biophysically by Bomben and Sontheimer (2008), who also showed that multinucleated glioma cells resulting from incomplete cell division during their extensive proliferation result in the functional loss of TRPC1 channels regulating calcium signaling during cytokinesis (Bomben and Sontheimer, 2010). These authors also provided *in vivo* evidence that loss of TRPC1 function impairs tumor growth in immunocompromised mice, suggesting that pharmacological inhibition of these channels may slow tumor growth (Bomben and Sontheimer, 2010).

##### TRPC3

TRPC3 levels are associated with both diagnostic and prognostic values: high-grade gliomas have higher TRPC3 expression levels than normal brain tissues, and glioma patients with high TRPC3 expression have a shorter survival time than patients with a lower TRPC3 expression (Chang et al., 2018). Reduced proliferation was demonstrated *in vitro* in U87MG glioma cells with a reduced expression of TRPC3 (Chang et al., 2018). Chang et al. (2018) showed, accordingly, that glioblastoma cell proliferation was decreased by ethyl-1-(4-2,3,3-trichloroacrylamide)phenyl)-5-(trifluoromethyl)-1H-pyrazole-4-carboxylate (Pyr3), a selective TRPC3 channel blocker (Figure 2). This compound induced caspase-dependent apoptosis and mitochondrial membrane potential imbalance in two glioblastoma cell lines as well as inhibition of migration and invasion *in vitro*; in a xenograft animal model *in vivo*, this compound in combination with temozolomide inhibited glioblastoma tumor growth (Chang et al., 2018).

##### TRPC6

Not only is the TRPC6 channel overexpressed in human glioma cells at both the protein and mRNA levels, as compared to normal glial cells (Chigurupati et al., 2010), but also TRPC6 expression relates to the grade of glioma (Ding et al., 2010). Hypoxia increases Notch 1 activation, which in turn induces the expression of TRPC6 in primary samples and cell lines derived from glioblastoma (Chigurupati et al., 2010). Under hypoxia, TRPC6 channels also control *in vitro* hydroxylation and stability of hypoxia inducible factor-1 alpha in human glioma cells (Li et al., 2015). Impairing TRPC6 activity *in vitro* in human glioma cells induced cell cycle arrest at the G2/M phase and, *in vivo*, reduced human xenograft growth in immunocompromised mice, while increasing the survival of the xenografted mice (Ding et al., 2010).

#### TRPV Channels

TRPV1 is the prototype of the TRPV channel family, which includes six members; it is activated by heat and synthetic or endogenous vanilloids (Caterina et al., 1997). TRPV2 shares approximately 50% sequence identity with TRPV1, while showing distinct cellular functions from those mediated by TRPV1 (Cohen et al., 2015). TRPV1 expression is mainly localized to the plasma membrane, while TRPV2 is localized to the intracellular membranes under unstimulated conditions (Cohen et al., 2013). TRPV2 is not stimulated by heat or by vanilloid exposure (Cohen et al., 2013 and 2015). TRPV2 activity is induced by 2-aminoethoxydiphenyl borate, probenecid, and cannabidiol and inhibited by ruthenium red, gadolinium, and tranilast (Caterina et al., 1997; Bang et al., 2007; Perálvarez-Marín et al., 2013; Nabissi et al., 2015). The translocation of TRPV2 from the endosome to the plasma membrane, a feature that influences both cell proliferation and cell death, is stimulated for example by growth factors, cytokines, hormones, and endocannabinoids (Liberati et al., 2014a,b). Uncontrolled cell proliferation and apoptotic resistance occur with loss or changes in TRPV2-mediated signals, whereas TRPV2 activation stimulates both the migration and the invasiveness of cancer cells (Liberati et al., 2014a,b).

The scientific literature reports the implications of TRPV1 and TRPV2 in glioma cell biology, as described below.

##### TRPV1

TRPV1 is mainly expressed by primary sensory neurons involved in nociception and neurogenic inflammation (Caterina et al., 2000).

TRPV1 gene and protein expressions are progressively lost, while the level of malignancy increases in gliomas, with a marked loss of TRPV1 expression in almost all (93%) of the glioblastomas analyzed by Amantini et al. (2007). Moreover, TRPV1 mRNA expression was correlated with patients’ overall survival (OS). TRPV1 variant 3 mRNA expression reached significance (*p* = 0.0009) for survival with short OS glioblastoma patients, showing a lower TRPV1 variant 3 mRNA expression compared with long OS patients (Nabissi et al., 2016). TRPV1 is implicated in the capsaicin-induced p38 mitogen-activated protein kinase-dependent apoptosis of glioma cells *in vitro* (Amantini et al., 2007). TRPV1 stimulation also triggers tumor cell death via the activating transcription factor-3 (ATF3)-controlled branch of the endoplasmic reticulum stress pathway.

Somatic mutant neural stem and precursor cells (NPCs) are thought to be the source of high-grade astrocytomas which are much more frequent in adults than in children. Stock et al. (2012) have shown that high-grade astrocytoma-associated NPCs induce tumor cell death via the release of endovanilloids, which induce Ca^2+^ responses. Endovanilloids directly stimulate the vanilloid receptor TRPV1 (Toth et al., 2009). However, the anti-tumorigenic response of NPCs is lost with age. Stock et al. (2012) report that NPC-mediated tumor suppression can be mimicked in the adult brain by systemic administration of the synthetic brain barrier-permeable vanilloid Arvanil (Figure 2), suggesting that TRPV1 agonists hold potential as new high-grade glioma therapeutics.

##### TRPV2

TRPV2 expression decreases during glioma progression to higher clinical stages; TRPV2 negatively controls glioma cell survival and proliferation and protects cells from Fas-induced apoptosis in an ERK-dependent manner (Nabissi et al., 2010). This receptor also negatively controls resistance to carmustine (BCNU)-induced apoptosis (Nabissi et al., 2010). High glioblastoma resistance to standard chemotherapy is one of the major hallmarks of glioblastoma biological aggressiveness. The TRPV2 agonist cannabidiol, by increasing TRPV2 expression and activity by triggering TRPV2-dependent Ca^2+^ influx, increases chemotherapeutic drug uptake and synergizes with cytotoxic agents (doxorubicin, temozolomide, and BCNU) to induce *in vitro* apoptosis in glioma cells but not in normal human astrocytes (Nabissi et al., 2013). TRPV2 activation promotes differentiation and inhibits the proliferation of glioblastoma stem cells (GSCs) *in vitro* and *in vivo* (Morelli et al., 2012). Redifferentiating cancer stem cell subpopulations could be achieved while using drug-induced differentiation, knowing that cancer stem cells are radio- and chemoresistant. Stimulating TRPV2 cannabidiol triggers GSC differentiation by inducing autophagy and inhibiting GSC proliferation and clonogenicity. Moreover, the cannabidiol and BCNU combination overcame GCS resistance to BCNU treatment by inducing apoptosis (Nabissi et al., 2015). In glioblastoma, TRPV2 is part of an interactome-based signature complex (Doñate-Macián et al., 2018), which is negatively associated with patient survival, and it is expressed in high risk of recurrence and temozolomide-resistant patients (Santoni et al., 2020a).

#### TRPML Channels

Endosome/lysosome Ca^2+^ channel proteins are characteristic of the TRPML channel family. In mammals, there are three TRPML proteins (TRPML-1, TRPML-2, and TRPML-3). A link between TRPML channel physiology and tumor biology has been suggested, and we focus here on glioma biology.

##### TRPML1

TRPML1, which is primarily localized in the late endosome/lysosome, is ubiquitously expressed in mammalian cells. It plays roles in the control of cell viability and in chaperone-mediated autophagy (Venkatachalam et al., 2006). A mutated TRPML1 gene in humans causes a neurodegenerative disease in children, i.e., mucolipidosis type IV (Bargal et al., 2000). TRPML1 is a proton-impermeable, cation-selective channel with permeability to both Ca^2+^ and Fe^2+^. Chaperone-mediated autophagy-related proteins [for example the heat shock cognate protein Hsc70 and the 40-kDa heat shock protein (Hsp40)] interact with the large TRPML1 intraluminal loop (Venkatachalam et al., 2006).

Morelli et al. (2019) showed that the loss/reduction of TRPML1 mRNA expression strongly correlates with short survival in glioblastoma patients. This feature could be explained, at least partly, by the fact that TRPML1 targets the apoptosis-like gene 2 (ALG-2) gene whose protein promotes caspase-3-independent cell death associated with glioblastoma progression and poor prognosis (Vergarajauregui et al., 2009; Zhang et al., 2017). Morelli et al. (2019) have also conducted elegant experiments with various glioma cell types to demonstrate that TRPML1 is an oxidative stress sensor that activates irreversible autophagy leading to cell death.

##### TRPML2

While TRPML2 is found in normal astrocytes and neural stem/progenitor cells, its expression at both mRNA and protein levels dramatically augment (Morelli et al., 2016) in high-grade glioblastoma cell lines of astrocytic origin and glioblastoma tissues (Morelli et al., 2016). Morelli et al. (2016) experimentally demonstrated that cell viability and proliferation are inhibited in TRPML2 knockdown glioblastoma cells, while caspase-3-dependent apoptosis is increased.

#### TRPM Channels

The TRPM subfamily is composed of eight members consisting of four six-transmembrane domain subunits, resulting in homomeric or heteromeric channels. TRPM subfamily members have been involved in several physiological functions and pathophysiological human processes.

##### TRPM2

Oxidative stress and tumor necrosis factor alpha are two extracellular signals known to activate TRPM2 channels, with consequently the activation of necrotic cell death (Zhang et al., 2006). Ishii et al. (2007) showed that the insertion of TRPM2 channels by means of transfection into the malignant glioma cell line A172 enhanced cell death induced by H_2_O_2_. In a recent study, Bao et al. (2020) observed a significant increase in TRPM2-AS, a long non-coding RNA with a length greater than 200 base pairs), which is transcribed from the antisense chain of TRPM2, in 111 glioma patients with glioma as compared to the normal control group. Overexpressing TRPM2-AS in human glioblastoma cells increases their proliferation, migration, and invasion, while downregulation of TRPM2-AS inhibits these three processes (Bao et al., 2020). TRPM2-AS signaling in glioma cells involves c-Jun N-terminal kinase (JNK), c-Jun protein, and regulator of G-protein signaling 4 (RGS4) (Bao et al., 2020).

##### TRPM7

A large number of breast, lung, pancreatic, prostate, gastric, and head and neck cancers and malignant gliomas express high TRPM7 levels (Jiang et al., 2007; Kim et al., 2008; Guilbert et al., 2009; Rybarczyk et al., 2012; Sun et al., 2014; Alptekin et al., 2015; Chen et al., 2016). For example, malignant glioma tissues express higher TRPM7 mRNA than normal brain tissues (Chen et al., 2016; Wan et al., 2020). TRPM7 silencing reduced glioma cell growth by inhibiting cell entry into S and G2/M phases and promoting cell apoptosis (Wan et al., 2020). TRPM7 expression in glioblastoma cells was found to be positively correlated with Notch1 signaling activity and CD133 and ALDH1 expression; briefly, downregulation of TRPM7 by siTRPM7 decreased Notch1 signaling whereas upregulation of TRPM7 increased Notch1 signaling (Wan et al., 2020).

Carvacrol (Figure 2) is one of the several inhibitors of TRPM7 already identified (Parnas et al., 2009). This compound is a secondary metabolite (a monoterpenoid phenol) found in oregano essential oils from numerous genera (Baser, 2008). Suppression of TRPM7 activity through the use of carvacrol and the use of TRPM7-siRNA dramatically reduced the proliferation, migration, and invasion levels of the U87MG malignant glioma cell line, which expresses higher levels of TRPM7 mRNA and protein than normal human astrocytes (Chen et al., 2015; Leng et al., 2015). MGR2 glioma cells also express TRPM7 and display TRPM7 currents (Chen et al., 2016). Chen et al. (2016) identified a widely used anesthetic compound in clinics since the 1970s, i.e., midazolam (Figure 2), as a TRPM7 inhibitor. The use of midazolam for *in vitro* treatment periods as short as seconds on glioma cells suppressed TRPM7 currents and calcium influx, while treatment for 48 h vanished TRPM7 expression (Chen et al., 2016). The inhibitory effect of midazolam on TRPM7 currents results in a decrease in proliferation and G0/G1 phase cell cycle arrest in two human glioblastoma cell lines (Chen et al., 2016). Of note, midazolam is a short-acting benzodiazepine that crosses the blood–brain barrier with a favorable pharmacological profile, and it could be used at first glance to treat patients with malignant glioma if one considers TRPM7 as a valuable target. However, the concentration (100 μM) of midazolam used *in vitro* in the study reported by Chen et al. (2016) is much higher than the clinical concentration ranges reported for this compound. It must indeed be emphasized that midazolam used at high doses induces sedative and hypnotic effects and therefore precludes its use as a chronic treatment for malignant glioma patients (Olkkola and Ahonen, 2008). Novel derivatives of midazolam or medical devices for its local delivery should be developed if it is to be used for glioma chemotherapy.

##### TRPM8

TRPM8 was first identified in prostate carcinoma (Tsavaler et al., 2001) and then in a number of other cancer types (Liu et al., 2014; Yee et al., 2014; Yu et al., 2014); it has been more recently shown to be upregulated in glioblastoma compared to normal brain tissue (Alptekin et al., 2015; Zeng et al., 2019), while TRPM8 expression is highly heterogeneous in human glioblastoma specimens as well as in established cell lines (Klumpp et al., 2017). Zeng et al. (2019) showed that high expression of TRPM8 mRNA was associated with a shorter OS time in patients with glioblastoma. TRPM8 channels facilitate Ca^2+^ entry in glioblastoma cells, and their activation has been shown to stimulate large-conductance K^+^ channel activity and, consequently, glioblastoma cell migration (Wondergem et al., 2008; Wondergem and Bartley, 2009; Klumpp et al., 2017). *In vitro*, using the U251 human glioblastoma cell line, Zeng et al. (2019) showed that TRPM8 enhances the sensitivity of glioblastoma cells to apoptosis and regulates the proliferation and invasion abilities. Klumpp et al. (2017) showed *in vitro* using human glioblastoma cells that (i) TRPM8 signaling is involved in cell cycle regulation and represses apoptotic cell death; (ii) clinically compatible ionizing radiation doses for treating glioblastoma patients induce upregulation of TRPM8 function; and (iii) elevated TRPM8 function, in turn, confers radioresistance (Klumpp et al., 2017). A combination of TRPM8 targeting and radiotherapy could be an interesting approach for future glioblastoma therapy. As developed in the next section, some strategies to target TRPM8 have already been developed and/or are ongoing.

## Therapeutic Perspectives

As summarized above, TRP channels exert various roles in cancer cell biology, including glioma ones (Gaunt et al., 2016; He and Ma, 2016; Jardin and Rosado, 2016; Li and Ding, 2017; Zhan and Shi, 2017). A number of more or less specific compounds from synthetic versus natural origin that selectively target different subtypes of TRP channels have been discovered, including some preclinical candidates (Wang et al., 2020). We recall these promising compounds below.

Some reasonably specific pharmacological TRPM8 inhibitors are already available (Ohmi et al., 2014; Lehto et al., 2015), including antibodies binding the extracellular TRPM8 protein and inhibiting TRPM8 function (Miller et al., 2014). However, the available studies have been performed *in vitro* only, and preclinical studies in orthotopic glioblastoma animal models are still missing. There is still a long road ahead until these types of compounds will enter clinics for treating malignant glioma patients.

Riluzole (Figure 2) is a TRPC5 agonist; however, it can also act on other ion channels so this limits its use. Riluzole is an approved drug for the treatment of amyotrophic lateral sclerosis, and it entered clinical trials for melanoma therapy. The precise mechanism(s) of action of riluzole is not yet fully deciphered. The riluzole-induced activation of TRPC5 channels, while expressed heterologously (as in HEK293 Human Embryonic Kidney cells) or endogenously (as in U87MG glioblastoma cells), seems to be independent of various cytosolic components, such as phospholipase C activity or intracellular calcium stores, suggesting therefore that riluzole could have a rather direct effect on TRPC5 (Richter et al., 2014). Furthermore, prednisolone (Figure 2), largely used in the context of glioma treatment to decrease glioblastoma-associated edema, also acts as a weak activator of TRPC5 (Beckmann et al., 2017). As emphasized above for TRPM8, there is still a long road before these types of compounds targeting TRPC5 will enter clinics to treat malignant glioma patients.

SKF-96365 (Figure 2), a non-specific TRPC6 and TRPC7 antagonist, displays cytotoxic effects in several cancer cell types (Song et al., 2014). In glioblastoma cells, SKF-96365 exerts anti-growth effects through the promotion of the reverse mode of Na^+^/Ca^2+^ exchangers, thereby increasing Ca^2+^ (Song et al., 2014). This compound does not seem very appropriate, at least in our current knowledge of its mode of action, to be an actual candidate to combat glioblastoma in clinical situations.

### Cannabinoids: New Application for Old Agents

In contrast to the compounds we refer to above, certain cannabinoids could be of major importance to combat glioblastoma in clinical situations and a TRP context as explained hereafter. The term cannabinoids originally described bioactive constituents of the plant *Cannabis sativa*, used traditionally for their medicinal purpose as well as their recreational properties. Cannabinoids can reduce glioma growth both *in vitro* and *in vivo* (Velasco et al., 2007; Sarfaraz et al., 2008). Among the cannabinoid compounds, we emphasize the potential of cannabidiol (Figure 2) to combat glioblastoma in clinical situations for the reasons we explained here below. Cannabidiol is a cannabinoid that lacks unwanted psychotropic liability and has no significant agonist activity on cannabinoid receptors (Howlett et al., 2002; Pertwee et al., 2005). Cannabidiol has been investigated as an antitumoral agent in a number of studies (Dumitru et al., 2018 for review).

*In vitro*, cannabidiol inhibits migration (Vaccani et al., 2005) and induces apoptosis in human glioma cells (Massi et al., 2004, 2006; Solinas et al., 2013), while it increases chemotherapeutic drug uptake and parallelly potentiates the cytotoxic activity of chemotherapeutic agents in a TRPV2-dependent manner in human glioma cells (Nabissi et al., 2013). *In vitro*, cannabidiol enhances the inhibitory effects of cannabinoid 1 and cannabinoid 2 receptor agonist delta(9)-tetrahydrocannabinol (Δ^9^-THC) on human glioblastoma cell survival and proliferation (Marcu et al., 2010). The combination of cannabidiol with Δ^9^-THC and temozolomide reduces the growth of U87MG glioma xenografts (Torres et al., 2011). Cannabidiol may also be effective at reducing the proliferation of GSC chemoresistant subpopulations present in glioblastomas (Singh et al., 2004). In glioma xenografts, including those derived from glioma stem cells, López-Valero et al. (2018) showed that a combined therapy of oral cannabinoids and temozolomide synergistically reduced the growth and enhanced the survival of xenografted animals. Cannabidiol, by activating TRPV2, (i) triggers GSC differentiation, (ii) activates their autophagic processes, (iii) inhibits glioma stem cell proliferation, (iv) inhibits their clonogenic capability, and (v) abrogates their resistance to carmustine (BCNU) (Nabissi et al., 2015).

A pilot phase I clinical trial for the treatment of glioblastoma patients indicated a good safety profile for Δ^9^-THC, which is a psychoactive cannabinoid (Velasco et al., 2007). The intratumoral administration of this compound was first tested in a small series of nine patients (Guzmán et al., 2006). Preclinical studies have also investigated the antitumor effects of the cannabinoid combination (Δ^9^-THC and cannabidiol) and found an enhanced antineoplastic effect (Ladin et al., 2016) in combination with temozolomide or radiotherapy (Torres et al., 2011; Scott et al., 2014; Ladin et al., 2016).

A placebo-controlled phase II clinical trial investigated a tetrahydrocannabidiol–cannabidiol mixture in combination with dose-intense temozolomide in glioblastoma (NCT01812603) (Schultz and Beyer, 2017). This study included 21 adult glioblastoma patients receiving a maximum of 12 sprays orally per day, delivering 100 μl of a solution containing 27 mg/ml Δ^9^-THC and 25 mg/ml cannabidiol. The control group received temozolomide and only reached a 44% 1-year survival rate. In sharp contrast, the tetrahydrocannabidiol–cannabidiol mixture plus temozolomide group showed an 83% 1-year survival rate, with a median survival of over 662 days compared with 369 days in the control group (Schultz and Beyer, 2017; Schultz, 2018). In another study, nine consecutive patients with brain tumors received cannabidiol at a daily dose of 400 mg concomitantly to the standard therapeutic procedure of maximal resection followed by combined radio- and chemotherapy and adjuvant chemotherapy (Likar et al., 2019). The authors reported that, by the time of the submission of their article, all but one patient were still alive, with a mean survival time of 22.3 months (range from 7 to 47). Importantly, the well-acknowledged median survival for glioblastoma patients is 16 months, as reported in the reference study by Stupp et al. (2009). A recent case report study demonstrated satisfactory clinical and imaging responses for two patients with a confirmed diagnosis of high-grade glioma (grades III or IV), benefiting after surgery from chemoradiation followed by the combination of procarbazine, lomustine, and vincristine associated with cannabidiol (Dall’Stella et al., 2019).

## Conclusion

Multiple ion channels rely on intracellular Ca^2+^, and it makes sense to target a common Ca^2+^ source, such as specific TRP channels, which are heavily involved in glioma cell biology.

We are only at the beginning of our understanding of the precise roles of various TRP channels in glioma cell biology, and further studies are required to truly understand the physiopathological roles of TRP channels in glioma progression. However, some promising data from the literature, even if still scarce, already point to the very high promise of targeting TRPV2 by means of cannabidiol, a cannabinoid that lacks unwanted psychotropic liability.

## Author Contributions

The author confirms being the sole contributor of this work and has approved it for publication.

## Conflict of Interest

The author declares that the research was conducted in the absence of any commercial or financial relationships that could be construed as a potential conflict of interest.

## Publisher’s Note

All claims expressed in this article are solely those of the authors and do not necessarily represent those of their affiliated organizations, or those of the publisher, the editors and the reviewers. Any product that may be evaluated in this article, or claim that may be made by its manufacturer, is not guaranteed or endorsed by the publisher.
